# Disruption of hepatic mitochondrial pyruvate and amino acid metabolism impairs gluconeogenesis and endurance exercise capacity in mice

**DOI:** 10.1152/ajpendo.00258.2023

**Published:** 2024-02-14

**Authors:** Michael R. Martino, Mohammad Habibi, Daniel Ferguson, Rita T. Brookheart, John P. Thyfault, Gretchen A. Meyer, Louise Lantier, Curtis C. Hughey, Brian N. Finck

**Affiliations:** ^1^Division of Nutritional Sciences and Obesity Medicine, Department of Medicine, Washington University School of Medicine, St. Louis, Missouri, United States; ^2^Department of Molecular & Integrative Physiology, University of Kansas Medical Center, Kansas City, Missouri, United States; ^3^Department of Medicine, Program in Physical Therapy, Washington University School of Medicine, St. Louis, Missouri, United States; ^4^Department of Molecular Physiology and Biophysics, Vanderbilt Mouse Metabolic Phenotyping Center, Vanderbilt University School of Medicine, Nashville, Tennessee, United States; ^5^Division of Molecular Medicine, Department of Medicine, University of Minnesota, Minneapolis, Minnesota, United States

**Keywords:** alanine transaminase, exercise, gluconeogenesis, mitochondrial pyruvate carrier

## Abstract

Exercise robustly increases the glucose demands of skeletal muscle. This demand is met by not only muscle glycogenolysis but also accelerated liver glucose production from hepatic glycogenolysis and gluconeogenesis to fuel mechanical work and prevent hypoglycemia during exercise. Hepatic gluconeogenesis during exercise is dependent on highly coordinated responses within and between muscle and liver. Specifically, exercise increases the rate at which gluconeogenic precursors such as pyruvate/lactate or amino acids are delivered from muscle to the liver, extracted by the liver, and channeled into glucose. Herein, we examined the effects of interrupting hepatic gluconeogenic efficiency and capacity on exercise performance by deleting mitochondrial pyruvate carrier 2 (MPC2) and/or alanine transaminase 2 (ALT2) in the liver of mice. We found that deletion of MPC2 or ALT2 alone did not significantly affect time to exhaustion or postexercise glucose concentrations in treadmill exercise tests, but mice lacking both MPC2 and ALT2 in hepatocytes (double knockout, DKO) reached exhaustion faster and exhibited lower circulating glucose during and after exercise. Use of ^2^H/^1^³C metabolic flux analyses demonstrated that DKO mice exhibited lower endogenous glucose production owing to decreased glycogenolysis and gluconeogenesis at rest and during exercise. Decreased gluconeogenesis was accompanied by lower anaplerotic, cataplerotic, and TCA cycle fluxes. Collectively, these findings demonstrate that the transition of the liver to the gluconeogenic mode is critical for preventing hypoglycemia and sustaining performance during exercise. The results also illustrate the need for interorgan cross talk during exercise as described by the Cahill and Cori cycles.

**NEW & NOTEWORTHY** Martino and colleagues examined the effects of inhibiting hepatic gluconeogenesis on exercise performance and systemic metabolism during treadmill exercise in mice. Combined inhibition of gluconeogenesis from lactate/pyruvate and alanine impaired exercise endurance and led to hypoglycemia during and after exercise. In contrast, suppressing either pyruvate-mediated or alanine-mediated gluconeogenesis alone had no effect on these parameters. These findings provide new insight into the molecular nodes that coordinate the metabolic responses of muscle and liver during exercise.

## Graphical abstract

**Figure:**
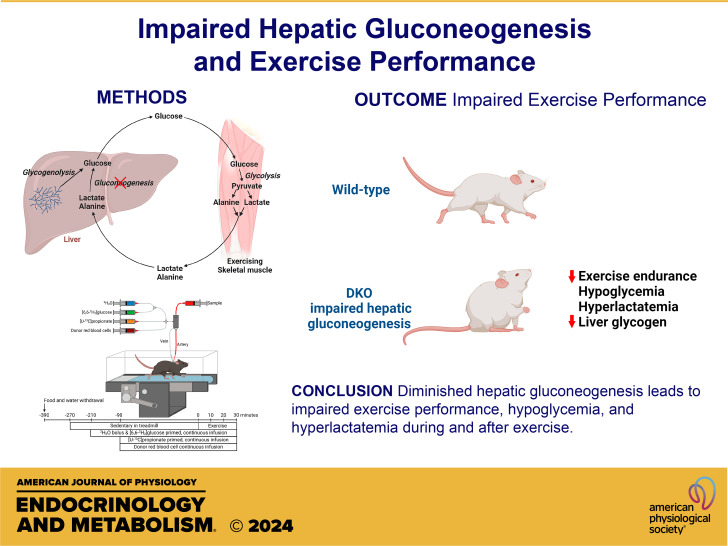

## INTRODUCTION

Exercise evokes dramatic changes in skeletal muscle and systemic metabolism that allow the organism to meet the energy demands of muscular work. Muscle glycogen serves to fuel muscle contraction, but muscle must also rely on blood glucose and other metabolic substrates to fuel muscle work. The liver is vital to the maintenance of adequate blood glucose concentrations during exercise and for replenishing muscle glycogen after exercise under fasting conditions (1, 2). Hepatic glucose output is the product of hepatic glycogenolysis and gluconeogenesis. Gluconeogenesis, the synthesis of new glucose from noncarbohydrate precursors (lactate/pyruvate, glycerol, and gluconeogenic amino acids), becomes increasingly important as liver glycogen levels are expended during prolonged exercise (2–4). Indeed, liver-specific deletion of cytosolic phosphoenolpyruvate carboxykinase (PEPCK1), which catalyzes a rate-limiting step in gluconeogenesis, leads to impaired exercise performance in mice (5).

### The Cori and Cahill Cycles

The stimulation of liver gluconeogenesis by exercise is dependent on highly coordinated metabolic responses within and between muscle and liver. It requires an increase in the rate at which gluconeogenic precursors are delivered from muscle to the liver, extracted by the liver, and channeled into glucose. Work by Drs. Gertrude and Carl Cori defined a cycle interorgan metabolic cross talk, which now bears their name (the Cori cycle), that is foundational for glucose metabolism under multiple conditions including exercise (6). During exercise, skeletal muscle anaerobic glucose metabolism and the production of pyruvate/lactate are elevated. Much of this lactate enters the bloodstream and is taken up by the liver for use as a gluconeogenic substrate. Glucose produced from this process can then be released by the liver and then travel through the blood to skeletal muscle to be metabolized via glycolysis.

The Cahill cycle is analogous in nature to the Cori cycle but involves shuttling alanine produced by peripheral tissues to the liver for its use in hepatic gluconeogenesis. Alanine release by muscle during exercise is increased (7) and alanine is present in blood far in excess of its proportional abundance to other amino acids in skeletal muscle (8, 9). Alanine can be generated from the breakdown of liver and muscle protein (10), but transamination of amino groups to pyruvate in muscle is likely a primary source of alanine; especially in the context of physiologic stimuli like exercise (11, 12). Muscle-synthesized alanine is delivered to the liver (7) where the amino group is removed and funneled to the urea cycle while re-synthesizing pyruvate, which can then be converted to glucose. Ultimately, the Cahill Cycle (13) serves two important functions during exercise; it delivers gluconeogenic precursors and muscle-derived amino groups in the nontoxic form of alanine to the liver.

### Molecular Regulators of Hepatic Gluconeogenesis

The Cori and Cahill cycles and their connection to hepatic gluconeogenesis have been well-studied. However, the regulatory nodes underlying their connection to hepatic gluconeogenesis have yet to be clearly defined. For example, the mitochondrial pyruvate carrier (MPC) is a heterodimeric inner mitochondrial membrane protein complex composed of two proteins (MPC1 and MPC2) that catalyze the mitochondrial import of pyruvate (14, 15). This step is critical for pyruvate entry into the gluconeogenic pathway because pyruvate carboxylase, which catalyzes a required step in pyruvate-mediated gluconeogenesis (16), is exclusively localized in the mitochondrial matrix. Mice lacking MPC proteins in hepatocytes are protected from hyperglycemia in diabetic mouse models due to reduced gluconeogenic flux (17, 18) and MPC inhibitors are also known to lower blood glucose in these models (19, 20). Similarly, alanine transaminase (ALT) enzymes are required for alanine to be converted to pyruvate and enter the gluconeogenic pathway via pyruvate carboxylase. Importantly, two distinct isozymes of ALT (ALT1 and ALT2) are encoded by two genes in Mammalia (*Gpt* and *Gpt2*). ALT1 is cytosolic whereas ALT2 is localized to the mitochondria (21–23). Recent work has demonstrated that hepatic ALT protein expression is increased in mice and humans with obesity and that suppressing the expression of ALT enzymes in liver has antidiabetic effects by reducing alanine-stimulated gluconeogenesis (24, 25). Herein, we tested the effects of liver MPC and ALT2 deletion on hepatic nutrient metabolism during exercise and its impact on exercise performance. Studies used mice deficient in hepatic MPC2, ALT2, or both MPC2 and ALT2 (double knockout, DKO) and evaluated their performance in exercise endurance and capacity tests. These indicators of exercise performance were accompanied by the quantification of in vivo glucose and associated nutrient fluxes at rest and during exercise.

## MATERIALS AND METHODS

### Animal Studies

All the experiments were performed with 8- to 16-wk-old mice of both sexes. At the conclusion of exercise experiments, mice were euthanized by injection of 150 mg/kg body wt of sodium pentobarbital “Fatal Plus” (Vortex Pharmaceuticals, Dearborn, MI) following the final blood glucose and lactate collection time point prior to tissue collection. For all other experiments, mice were euthanized with CO_2_. All the experiments involving mice were approved by the Institutional Animal Care and Use Committees of Washington University in St. Louis and Vanderbilt University and are in agreement with the Guide for the Care and Use of Laboratory Animals.

### Generation of DKO Mice

The generation of LS-*Mpc2^−^*^/−^ and LS-*Gpt2^−^*^/−^ mice has been described previously (18, 24). *Gpt2* fl/fl mice were mated with LS-*Mpc2^−^*^/−^ mice to generate LS-*Gpt2/Mpc2*^−/−^ (DKO) mice (26, 27). Littermate mice not expressing Cre (fl/fl mice or double *Mpc2*/*Gpt2* fl/fl mice) were used as control mice in all experiments.

### Exercise Protocol

**Figure 1.**
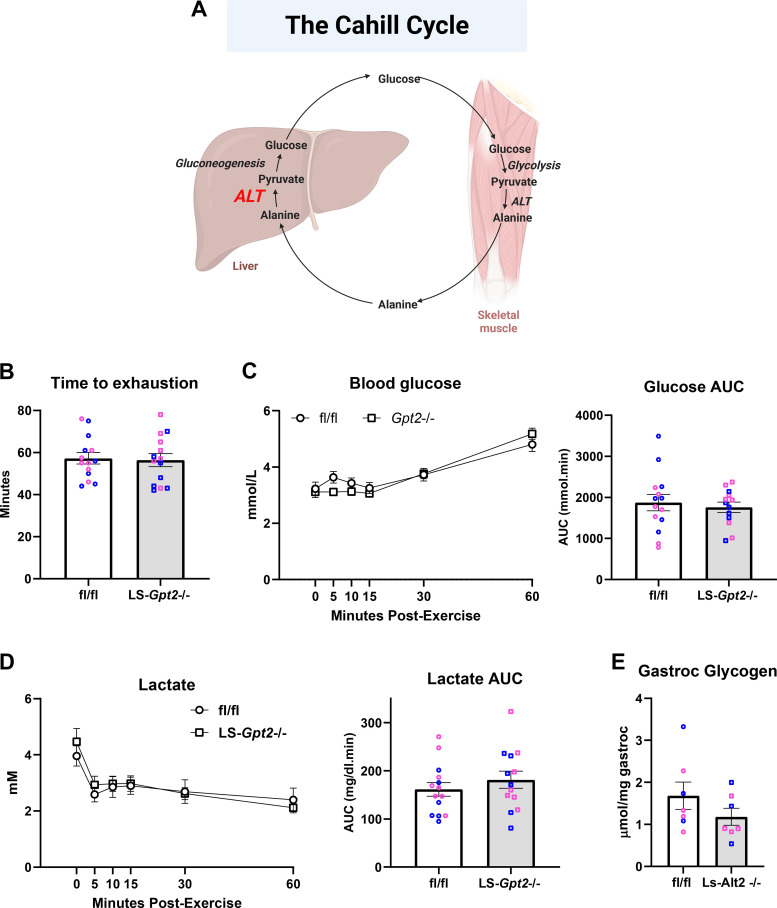
*A*: schematic depiction of the Cahill cycle, wherein skeletal muscle derived alanine is converted to glucose in the liver. Created using BioRender.com. *B*: time taken to reach exhaustion during a graded exercise test in fl/fl and LS-*Gpt2^−^*^/−^ mice. Blood glucose (*C*) and blood lactate (*D*) concentrations and area under the curve (AUC) in fl/fl and LS-*Gpt2^−^*^/−^ mice during the 60-min postexercise recovery period. For *B*–*D*, *n* = 14 mice for each genotype (*n* = 7 mice each sex within genotypes). *E*: gastrocnemius glycogen in fl/fl and LS-*Gpt2^−^*^/−^ mice at 60-min postexercise. *n* = 7 mice for each genotype (*n* = 4 female and 3 male mice of each genotype). Data are presented as means ± SE. Statistical significance was calculated using an unpaired Student’s *t* test. Pink data points indicate female mice and blue data points indicate male mice. ALT, alanine transaminase.

**Figure 2.**
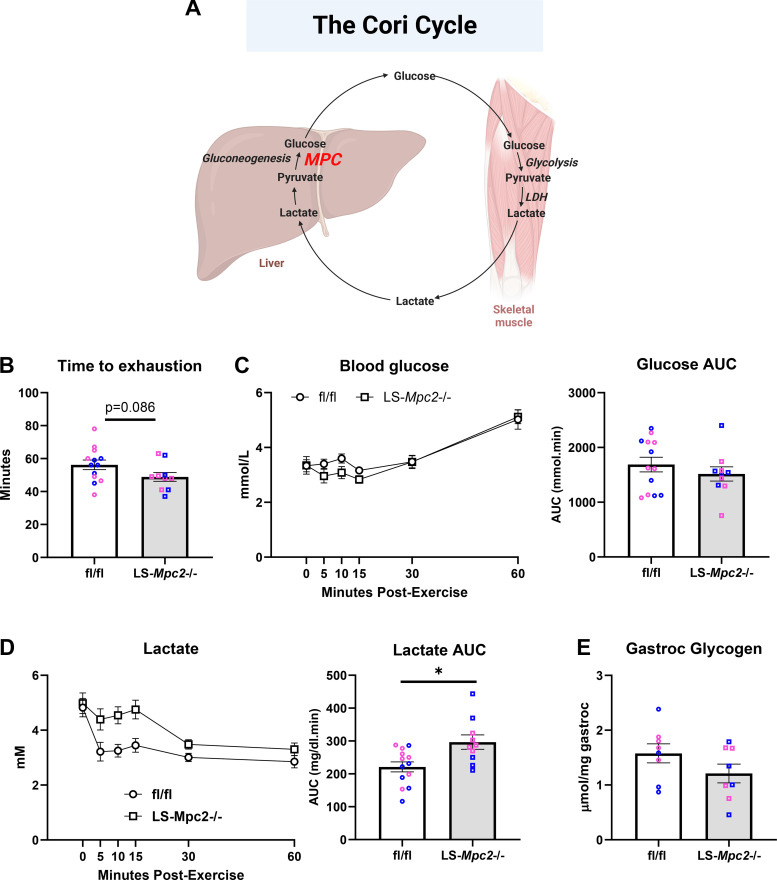
*A*: schematic depiction of the Cori cycle, wherein skeletal muscle derived lactate is converted to glucose in the liver. Created using BioRender.com. *B*: time taken to reach exhaustion during a graded exercise test in fl/fl and LS-*Mpc2^−^*^/−^ mice. Blood glucose (*C*) and blood lactate (*D*) concentrations and area under the curve (AUC) in fl/fl and LS-*Mpc2^−^*^/−^ mice during the 60-min postexercise recovery period. For *B*–*D*, *n* = 7 fl/fl female, *n* = 5 fl/fl male, *n* = 6 LS-*Mpc2^−^*^/−^ female, and *n* = 4 LS-*Mpc2^−^*^/−^ male mice). *E*: gastrocnemius glycogen in fl/fl and LS-*Mpc2^−^*^/−^ mice at 60-min postexercise. *n* = 8 mice for each genotype (*n* = 4 each sex within genotypes). Data are presented as means ± SE. *P < 0.05 vs. fl/fl control. Statistical significance was calculated using an unpaired Student’s *t* test. Pink data points indicate female mice and blue data points indicate male mice. LDH, lactate dehydrogenase; MPC, mitochondrial pyruvate carrier.

For exercise studies shown in Figs. 1, 2, and 4, mice were run to exhaustion on a closed treadmill (Columbus Instruments) with a shock grid that delivered a mild electrical stimulus (16–28 V) to encourage continuous running. Food was removed 4.5 h before exercise, bedding was replaced with aspen chip bedding, and initial body weights were recorded. Mice were weighed immediately before the run and then acclimated to the treadmill with 0° incline at 0 m/min for 5 min. The speed was then increased to 5 m/min and maintained for 5 min. After 5 min of continuous running, the speed was increased to 10 m/min, after 10 min, the speed was increased to 15 m/min, after 20 min, the speed was increased to 25 m/min, and after 35 min the speed was increase to 30 m/min and maintained until the mice reached exhaustion. Exhaustion was determined by the refusal of mice to remain on the treadmill belt for 10 s and lack of movement upon removal from the treadmill. Blood glucose and lactate were measured with a One-Touch Ultra glucometer (LifeScan) and a Lactate Plus lactate meter (NovaBiomedical), respectively, from a single drop of blood from the tail vein. Blood glucose and lactate concentrations were measured immediately upon exhaustion (*T* = 0), and at *T* = 5, 10, 15, 30, and 60 min postexhaustion. Mice remained fasted during the 1-h postexercise period.

### Exercise Protocol

Prior to studies shown in Fig. 5 and 6, mice were transferred to the Vanderbilt University Mouse Metabolic Phenotyping Core. After 4 wk of quarantine and acclimation, mice underwent an exercise stress test to assess maximal running speed. In this paradigm, the treadmill speed starts at 10 m/min and the speed increases 4 m/min every 3 min until exhaustion. Thereafter, catheters were implanted in the jugular vein and carotid artery for stable isotope infusion and sampling protocols as previously described (5, 28, 29). The free ends of the catheters were tunneled under the skin to the back of the neck and the exteriorized ends of the vascular catheters were flushed with 200 U/mL of heparinized saline and sealed with stainless-steel plugs. Following the surgical procedures, mice were individually housed and provided ∼9–10 days of postoperative recovery prior to stable isotope infusions studies during rest and acute exercise.

The weights of all mice were within 10% of presurgery body weight prior to the stable isotope infusions and the exercise protocol. Food and water were withdrawn within 1 h of the start of light cycle (6:00–8:00 AM). Two hours into the fast, mice were placed in an enclosed single-lane treadmill (Columbus Instruments, Columbus, OH) and the exteriorized catheters were connected to infusion syringes (Fig. 4). Three hours into the fast, an 80 μL arterial blood sample was obtained to determine natural isotopic enrichment of plasma glucose. Immediately following this sample, stable isotope infusions were initiated as previously performed (5, 29) (Fig. 5). Briefly, a ^2^H_2_O (99.9%)-saline bolus containing [6,6-^2^H_2_]glucose (99%) was administered over 25 min to both enrich body water and deliver a [6,6-^2^H_2_]glucose prime (440 μmol/kg). This was immediately followed by a continuous infusion of [6,6-^2^H_2_]glucose (4.4 μmol/kg/min). A primed (1.1 mmol/kg), continuous (0.055 mmol/kg/min) intravenous infusion of [U-^13^C]propionate was started 2 h after the ^2^H_2_O bolus and [6,6-^2^H_2_]glucose prime. Four 100–150 µL arterial blood samples were obtained (90–120 min following the [^13^C_3_] propionate bolus) to determine arterial blood glucose concentration as well as plasma glucose enrichment used in ^2^H/^13^C metabolic flux analyses protocols to quantify hepatic glucose and associated nutrient fluxes. The sample taken at 90 min following the [U-^13^C]propionate bolus (Time = 0 min) was obtained while mice were at rest on a stationary treadmill. Samples taken 100–120 min following the [U-^13^C]propionate bolus (Time = 10–30 min) were obtained while mice were performing a 30-min acute treadmill running bout at 12 m/min. Donor red blood cells were given by constant rate infusion for the duration of the study to ensure hematocrit did not fall more than 10%. Immediately following the exercise bout, mice were euthanized via pentobarbital and tissue collected was flash frozen.

### Glucose Derivatizations and GC-MS

Forty microliters of plasma from the −210, 0-, 10-, 20-, and 30-min time points was processed to obtain aldonitrile, methyloxime, and di-*O*-isopropylidene derivatives of glucose developed and described elsewhere (30, 31). The GC-MS injection volume of glucose derivatives was 1 µL with purge flow times between 20 and 120 s. GC-MS was completed using an Agilent 7890A gas chromatograph with an HP-5 ms capillary column (Agilent J&W Scientific) coupled to an Agilent 5975C mass spectrometer. For aldonitrile and di-*O*-isopropylidene derivatives, the column temperature was 80°C for 1 min, ramped at 20 °C/min up to 280°C where it was held for 4 min, and then ramped at 40 °C/min to 325°C. For the methyloxime derivatives, the column temperature was 80°C for 1 min, ramped to 280°C at 10 °C/min where it was held for 4 min, and subsequently ramped to 325°C at 40 °C/min. The MS was run in scan mode for *m*/*z* 100–500 for aldonitrile derivatives, *m*/*z* 140–260 for methyloxime derivatives, and *m*/*z* 300–320 for di-*O*-isopropylidene derivatives. All sample derivatives from each time point were run in duplicate. A custom MATLAB function was used to integrate derivative peaks to obtain mass isotopomer distributions (MIDs) for six glucose fragment ions. The following fragment ion ranges were used for determining uncorrected MIDs: aldonitrile, *m*/*z* 173–178, 259–266, 284–291, and 370–379; methyloxime, *m*/*z* 145–149; di-O-isopropylidene, *m*/*z* 301–314. The use of six fragment ions from three glucose derivatives provides increased information related to the labeling pattern of glucose (see Supplemental Table S1; all Supplemental material is available at https://doi.org/10.6084/m9.figshare.25035806.v1). Measurement accuracy was evaluated by comparing theoretical MID computed from the known naturally occurring isotope abundances and experimental values of control samples that were unenriched.

### ^2^H/^1^³C Metabolic Flux Analysis

The ^2^H/^1^³C metabolic flux analysis approach quantifies glucose production and associated nutrient fluxes using Isotopomer Network Compartmental Analysis (INCA) software [accessible at http://mfa.vueinnovations.com/mfa; (32)], which is based on the elementary metabolite units (EMU) framework (33). Using INCA, a metabolic reaction network that includes the hydrogen and carbon atom transitions for reactions in glucose-producing and TCA cycle pathways are defined (Supplemental Table S2). This information enumerates isotopomer and mass balances that detail the conservation of hydrogen and carbon atoms in the network. The reaction network used in this study has been thoroughly described (31) and used to quantify glucose and TCA cycle fluxes previously (29, 34–36). After constraining flux through citrate synthase to an arbitrary value of 100, relative fluxes within the reaction network are estimated simultaneously by regressing the model in an iterative manner to fit the raw mass isotopomer data obtained from the glucose fragments. This involves an optimization search to identify flux parameters that minimize the sum-of-squared residuals (SSR) between the simulated and experimental measurements (32, 37) (Supplemental Table S3). Flux estimates were repeated 50 times from random initial values, goodness of fit was evaluated by a χ^2^ test (*P* = 0.05), and confidence intervals of 95% were determined. Absolute fluxes were determined from relative fluxes using the known infusion rate of [6,6-^2^H_2_]glucose and mouse body weights.

### Muscle Glycogen Quantification

Skeletal muscle tissue was collected at euthanasia and snap frozen in liquid nitrogen. Glycogen concentration was determined using frozen tissues (30–90 mg) hydrolyzed in 0.3 mL of 30% (wt/vol) KOH solution in a boiling water bath for 30 min. At 10 and 20 min of the incubation, tubes were shaken by hand to facilitate the digestion. After cooling to room temperature, 0.1 mL of 1 M Na_2_SO_4_ and 0.8 mL of ethanol were added, the samples were boiled again for 5 min to facilitate precipitation of glycogen and then centrifuged at 10,000 *g* for 5 min. The glycogen pellet was dissolved in 0.2 mL of water, and two additional ethanol precipitations were performed. The final pellet was dried and dissolved in 0.2 mL of 0.3-mg/mL amyloglucosidase in 0.2 M sodium acetate buffer (pH 4.8) and incubated for 3 h at 40°C. The reaction mixture was diluted two- to fivefold with water. To determine the glucose concentration, 5 mL of the diluted sample was added to 0.2 mL of the glucose assay solution which contains 0.3 M triethanolamine–KOH (pH 7.5), 1 mM ATP, 0.9 mM b-NADP, and 5 mg of G-6P dehydrogenase per mL. The absorbance at 340 nm was determined before and after addition of 1 mg of hexokinase. Glycogen content is expressed as micromoles of glucosyl units per gram (wet weight) (38).

### Protein Isolation and Western Blotting

For Western blotting, liver lysates were collected in RIPA lysis buffer (Cell Signaling Technology; 9806) with protease/phosphatase inhibitors (Cell Signaling Technology; 5872) using a TissueLyser. Lysates were normalized to protein concentration, denatured, and run on NuPAGE precast 4–12% gels (Thermo Fisher Scientific) using MOPS or MES buffers (Thermo Fisher Scientific). Separated proteins were transferred to Immobilon PVDF membrane and blocked for 1 h with 5% BSA in Tris-buffered saline-Tween 20 (TBST). The antibodies used in this study were ALT1 (Abcam; ab154034, 1:1,000), ALT2 (Abcam; ab202083, 1:1,000), MPC1 (Cell Signaling Technology; D2L9I, 1:1,000), MPC2 (Cell Signaling Technology; D4I7G, 1:1,000), Vinculin (Cell Signaling Technology; 13901, 1:1,000), PEPCK (Cayman Chemical; 10004943, 1:1,000), pyruvate carboxylase (PC) (Cell Signaling Technology; 66470, 1:1,000), citrate synthase (CS) (Cell Signaling Technology; 14309, 1:1,000), lactate dehydrogenase (LDH) (Abcam; ab52488, 1:1,000), and OXPHOS cocktail (Abcam; ab110413, 1:1,000). The secondary antibodies including Goat anti-Rabbit (IRDye 800CW Goat anti-Rabbit IgG Secondary Antibody, LI-COR, 926-32211) and Goat anti-mouse (IRDye 680RD Goat anti-Mouse IgG Secondary Antibody, LI-COR, 926-68070) were used at a 1:10,000 dilution. Blots were imaged using a LI-COR Odyssey system and quantified with Image Studio Lite software. All antibodies were obtained from commercial sources that have validated the antibodies for specificity. When possible, we have internally validated antibodies through use of knockout tissues as shown in the figures herein. Supplemental uncut Western blot images can be found at https://doi.org/10.6084/m9.figshare.25035806.v1.

### Hepatocyte Isolation

Hepatocytes were isolated by perfusing livers of anesthetized mice with DMEM media containing collagenase from *Clostridium histolyticum* (Sigma Chemical Co.) as reported previously (18). Briefly, hepatocytes were plated overnight in collagen-coated 12-well plates (200,000 cells/mL) with DMEM containing 10% FBS plus an antibiotic cocktail (penicillin/streptomycin and amphotericin) and washed twice the following morning with glucose-free Hank’s balanced salt solution (GF-HBSS) containing 127 mM NaCl, 3.5 mM KCl, 0.44 mM KH_2_PO_4_, 4.2 mM NaHCO_3_, 0.33 mM Na_2_HPO_4_, 1 mM CaCl_2_, 20 mM HEPES, pH 7.4.

### Glucose Production Assay

Following the final wash, hepatocytes were starved for 2 h in GF-HBSS. HBSS was removed, cells were washed in fresh HBSS, and then treated for 3 h in HBSS containing glucagon (100 ng/mL) alone or 5 mM sodium pyruvate or 20 mM alanine. After the 3-h incubation, media was collected and glucose concentrations were measured using a glucose oxidase-based glucose assay kit (MilliporeSigma; Cat. No. GAGO20). Glucose concentrations were normalized to cell protein amount measured by Micro BCA kit (Thermo Fisher).

### Statistical Analyses

Figures were prepared using Prism version 8.0.1 for Windows (GraphPad Software, La Jolla, CA, www.graphpad.com). All data are presented as the means ± SE. Statistical significance was calculated using an unpaired Student’s *t* test or two-way analysis of variance (ANOVA) with repeated measures for data analysis, with a statistically significant difference defined as *P* ≤ 0.05.

## RESULTS

### Loss of Liver ALT2 or MPC2 Alone Does Not Limit Exercise Performance

The conversion of alanine to glucose by the liver is believed to be important to maintaining blood glucose concentrations during and after exercise when food is not available via the Cahill Cycle (Fig. 1*A*). We recently generated mice with liver-specific deletion of *Gpt2*, which encodes the mitochondrial alanine transaminase ALT2, and have reported their phenotype at baseline and after prolonged fast (24). Briefly, these mice are overtly normal, exhibit no differences in body weight, nor did they exhibit any defects in gluconeogenesis or hypoglycemia under the fasting conditions. To determine the contribution of hepatic amino acid metabolism to exercise performance and metabolism, we subjected LS-*Gpt2^−^*^/−^ mice to an acute, graded-intensity treadmill exercise challenge that was designed to evaluate exercise endurance. Compared with littermate wild-type (WT) mice, male and female LS-*Gpt2^−^*^/−^ mice ran for a similar duration and distance (Fig. 1*B*). After exercise, fasted blood glucose and lactate concentrations were similar between genotypes during the 1-h recovery period (Fig. 1, *C* and *D*) and there was no difference in skeletal muscle glycogen content at the end of the 1-h recovery period (Fig. 1*E*). This indicates that loss of ALT2 in liver does not affect exercise performance or recovery in the paradigm and parameters studied.

We also determined whether suppression of mitochondrial pyruvate metabolism in the liver could affect exercise performance given that lactate-/pyruvate-supported gluconeogenesis by the liver is believed to be important via the Cori cycle (Fig. 2*A*). LS-*Mpc2^−^*^/−^ mice have been previously characterized (18) and exhibit no over phenotype with regards to body weight or circulating glucose or other parameters in the fed state. We have demonstrated that loss of MPC2 protein destabilizes the MPC complex essentially resulting in a double knockout for MPC2 and its heterodimeric partner MPC1 (18). We found that time to exhaustion in the graded treadmill exercise paradigm was not different between LS-*Mpc2^−^*^/−^ and WT littermate male and female mice (Fig. 2*B*), though LS-*Mpc2^−^*^/−^ mice tended to run for shorter times. WT and LS-*Mpc2^−^*^/−^ mice had similar blood glucose concentrations during the 1-h recovery period (Fig. 2*C*), but the lactate area under the curve (AUC) was 34% greater in LS-*Mpc2^−^*^/−^ mice (Fig. 2*D*) likely reflecting impaired Cori cycling. Gastrocnemius glycogen content was similar between WT and LS-*Mpc2^−^*^/−^ mice at the end of the recovery period (Fig. 2*E*), again suggesting that glycogen replenishment from blood glucose was unimpaired by loss of MPC in the liver. Thus, loss of the MPC in liver did not affect exercise tolerance or recovery.

### Concomitant Loss of Liver ALT2 and MPC2 Impairs Endurance Exercise Performance

**Figure 3.**
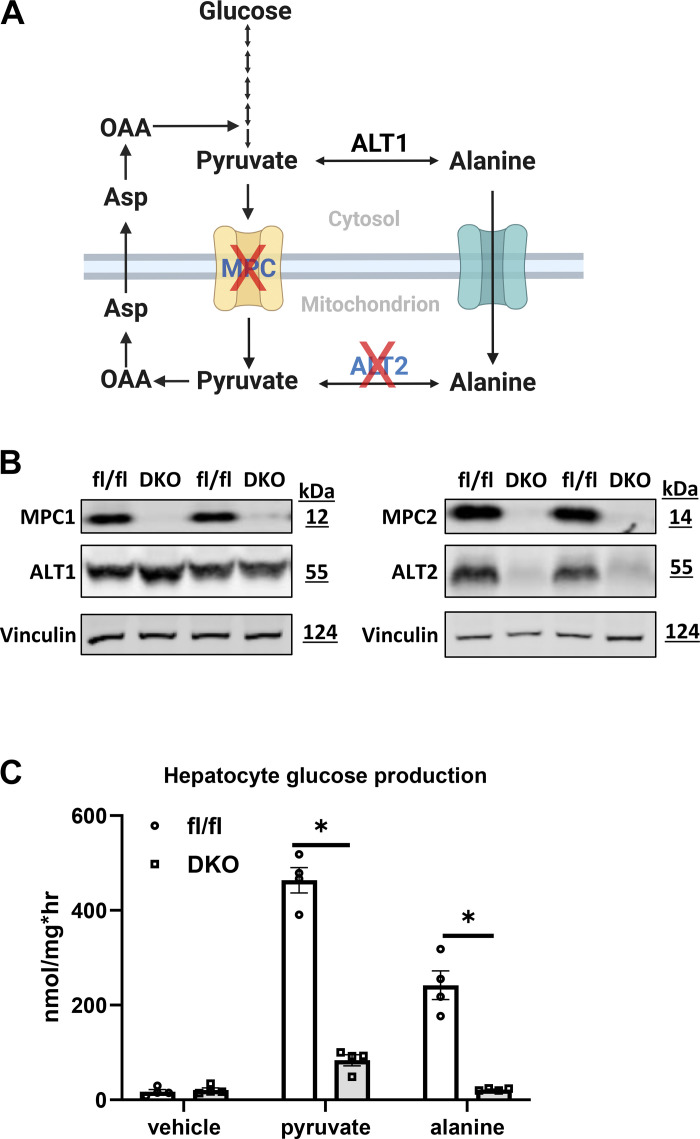
*A*: schematic representation of gluconeogenesis from pyruvate and alanine and the potential impact of deleting the mitochondrial pyruvate carrier (MPC) and alanine transaminase 2 (ALT2) enzymes. Created using BioRender.com. *B*: representative Western blots using liver protein lysates from fl/fl and double knockout (DKO) mice using the indicated antibodies. *C*: glucose concentrations in the media of cultured hepatocytes stimulated with glucagon in the presence of gluconeogenic substrates. Pyr, pyruvate (5 mM); Ala, alanine (20 mM). Data are presented as means ± SE. *n* = 4 technical replicates each group. *P < 0.05 vs. fl/fl control. Statistical significance was calculated using an unpaired Student’s *t* test.

We crossed LS-*Gpt2^−^*^/−^ mice with LS-*Mpc2*^−/−^ mice to generate double knockout (DKO) mice with liver-specific deletion of both ALT2 and MPC2 (39). This blocks both pathways for alanine and pyruvate entry into gluconeogenic pathways (Fig. 3*A*). Western blotting analyses confirmed that ALT2, MPC2, and MPC1 were absent from the livers of DKO mice, but that ALT1 was still expressed (Fig. 3*B*). To determine the hepatocyte-intrinsic effects of DKO on glucose production, hepatocytes were isolated from littermate WT and DKO mice and glucagon-stimulated glucose production in response to pyruvate or alanine was assessed. As predicted, hepatocytes from the DKO mice produced significantly less glucose when supplied with pyruvate or alanine as a gluconeogenic substrate (Fig. 3*C*).

**Figure 4.**
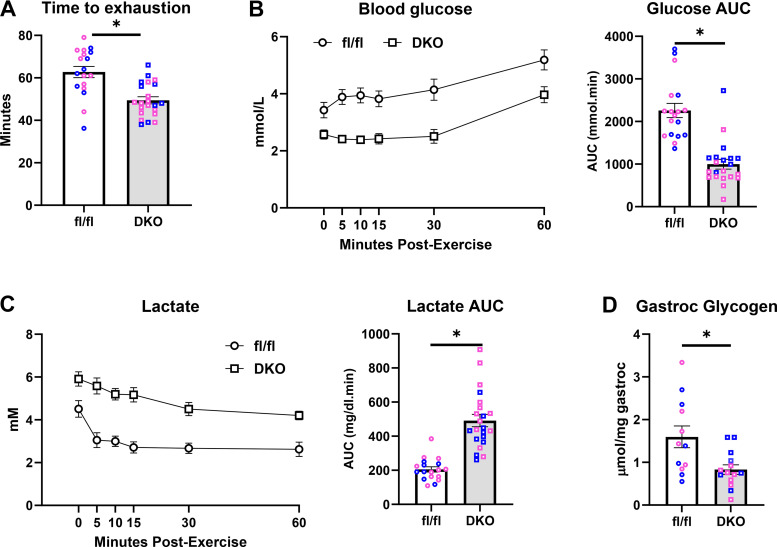
*A*: exercise time to exhaustion in fl/fl and double knockout (DKO) mice. Blood glucose (*B*) and blood lactate (*C*) concentrations and area under the curve (AUC) in fl/fl and DKO mice during the 60-min postexercise recovery period. For *A*–*C*, *n* = 10 fl/fl female mice, *n* = 8 fl/fl male mice, *n* = 13 LS-DKO female mice, and *n* = 9 LS-DKO male mice. *D*: glycogen concentrations of gastrocnemius muscles taken 60-min postexercise in fl/fl and DKO mice. *n* = 6 fl/fl female, *n* = 6 fl/fl male, *n* = 7 LS-DKO female, and *n* = 7 LS-DKO male mice. Data are presented as means ± SE. Pink data points indicate female mice and blue data points indicate male mice. *P < 0.05 vs. fl/fl control. Statistical significance was calculated using an unpaired Student’s *t* test.

We next evaluated the time to exhaustion in the graded treadmill exercise paradigm and found that, compared with WT littermate controls, male and female mice lacking both ALT2 and MPC2 in liver exhibited a significant reduction in time to exhaustion (Fig. 4*A*). Furthermore, DKO mice exhibited significantly lower blood glucose concentrations postexercise as indicated by a reduction in fasted glucose AUC compared with WT controls during the 1-h recovery period (Fig. 4*B*) whereas lactate AUC during recovery was increased during the same time (Fig. 4*C*). Finally, DKO mice also had significantly less glycogen in gastrocnemius muscle compared with littermate controls after 1 h of recovery from exercise (Fig. 4*D*). This finding could suggest that lower liver glucose production and blood glucose drives muscle to rely on its glycogen stores for glucose demands and/or that impaired gluconeogenesis and lower blood glucose concentrations were impacting the replenishment of muscle glycogen.

### Liver-Specific DKO Mice Are Hypoglycemic during Exercise with Impaired Anaplerotic and Cataplerotic Metabolism

**Figure 5.**
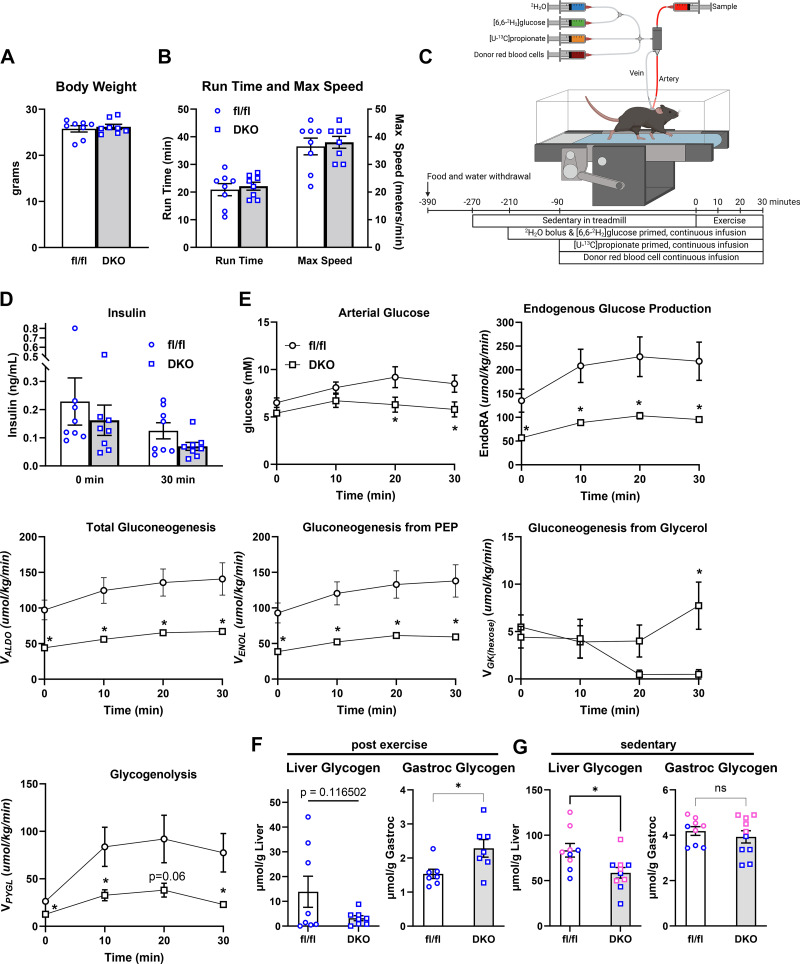
Body weight (*A*), run time, and maximal running speed (*B*) of wild type (WT) and double knockout (DKO) in an acute exercise stress test (*n* = 8 mice each genotype). After 4 wk of quarantine and acclimation at Vanderbilt University, surgically naïve mice underwent an exercise stress test to assess maximal running speed. In this paradigm, the treadmill speed starts at 10 m/min and the speed increases 4 m/min every 3 min until exhaustion.*P < 0.05 vs. fl/fl by unpaired Student’s *t* test. *C*: exercise experiment schematic. Created using BioRender.com. Stable isotope infusions at rest and during acute treadmill running bout were performed in mice ∼9–10 days following carotid arterial and jugular catheter implantation surgeries. At 210 min prior to the treadmill running bout (3 h of fasting), a ^2^H_2_O bolus was administered into the venous circulation to enrich total body water at 4.5%. A [6,6-^2^H_2_]glucose prime was infused followed by a continuous infusion was initiated with the ^2^H_2_O bolus. Ninety minutes before the onset of exercise (5 h of fasting), a primed, continuous infusion of [U-13C]propionate was started. Donor red blood cells were administered to prevent a decline in hematocrit. Arterial samples were obtained prior to stable isotope infusion as well as during 30-min exercise bout for ^2^H/^13^C metabolic flux analysis. *D*: plasma insulin concentrations at baseline (0 min) and after 30 min of exercise. *E*: a time course of blood glucose concentration (mmol/L) in fl/fl and DKO mice prior to (0-min time point) and during a 30-min treadmill run (10–30 min time points). Model-estimated, nutrient fluxes (µmol/kg/min) in fl/fl and DKO mice prior to and during a 30-min of treadmill run for endogenous glucose production, total gluconeogenesis (*V*_Aldo_), gluconeogenesis from phosphoenolpyruvate (*V*_Enol_), gluconeogenesis from glycerol (*V*_GK_), and glycogenolysis (*V*_PYGL_). *P < 0.05 vs. fl/fl at specified time point by two-way repeated-measures ANOVA followed by Šidák’s post hoc tests. *F*: liver and gastrocnemius glycogen content in mice euthanized immediately at the end of the studies shown in *A*–*E* (*n* = 8 mice each genotype). *P < 0.05 vs. fl/fl by unpaired Student’s *t* test. *G*: liver and gastrocnemius glycogen content in sedentary mice euthanized after a 4-h fast (*n* = 9 fl/fl and *n* = 10 DKO mice). *P < 0.05 vs. fl/fl by unpaired Student’s *t* test. Pink data points indicate female mice and blue data points indicate male mice.

To evaluate liver metabolism during treadmill exercise, a separate cohort of male mice were shipped to the Vanderbilt Mouse Metabolic Phenotyping Core for further evaluation. After 4 wk of quarantine and acclimation, mice underwent an exercise stress test to assess maximal running speed. In this paradigm, the treadmill speed starts at 10 m/min and the speed increases 4 m/min every 3 min until exhaustion. As expected, body weight of these mice was not different (Fig. 5*A*). Despite the striking defects in gluconeogenesis, time to exhaustion and maximal running speed in an exercise stress test (analogous to a V̇o_2max_ test) in the DKO mice was not different than WT control mice (Fig. 5*B*). Mice were then surgically catheterized to allow for continued sampling of blood during exercise to directly assess gluconeogenesis by using ^2^H/^13^C metabolic flux analysis by infusing [6,6-^2^H_2_]glucose,^2^H_2_O, and [U-^13^C]propionate as previously described (5) and as shown in Fig. 5*C*.

After recovery, mice underwent a 30-min acute treadmill running bout at 12 m/min. The slower speed and duration were chosen so that mice of both genotypes could complete the entire exercise bout. Thirty minutes of exercise tended to decrease plasma insulin concentrations compared with baseline (exercise effect: *P* = 0.07), but insulin concentrations were not affected by genotype at either time point (Fig. 5*D*). Although DKO mice exhibited normal glucose concentrations at the start of the exercise test and after 10 min of running, blood glucose concentrations were lower compared with WT controls after 20 and 30 min of treadmill exercise (Fig. 5*E*). Consistent with the observed hypoglycemia, quantification of nutrient fluxes revealed that endogenous glucose production, total gluconeogenesis, and gluconeogenesis from phosphoenolpyruvate (PEP) were significantly reduced in DKO mice compared with WT controls at the start of the exercise bout and at all times during exercise (Fig. 5*E*). Interestingly, gluconeogenesis from glycerol was not affected by loss of MPC and ALT2 activity at baseline, and was actually increased in DKO mice versus WT controls after 30 min of exercise (Fig. 5*E*). Finally, glucose production from glycogenolysis, which should not be directly impacted by loss of the MPC and ALT2, was markedly reduced in DKO mice compared with WT controls at every time point (Fig. 5*E*).

Therefore, we analyzed liver and muscle glycogen in these mice at the end of the experiment. We found that liver glycogen content tended to be diminished in whereas muscle glycogen was significantly increased in DKO mice at the end of the experiment (Fig. 5*F*). We also assessed glycogen content in a cohort of sedentary mice that had been fasted for 4 h (similar to exercised mice) and found that liver glycogen was significantly diminished in sedentary DKO mice compared with WT controls (Fig. 5*G*). There was no effect of genotype on muscle glycogen in sedentary mice.

**Figure 6.**
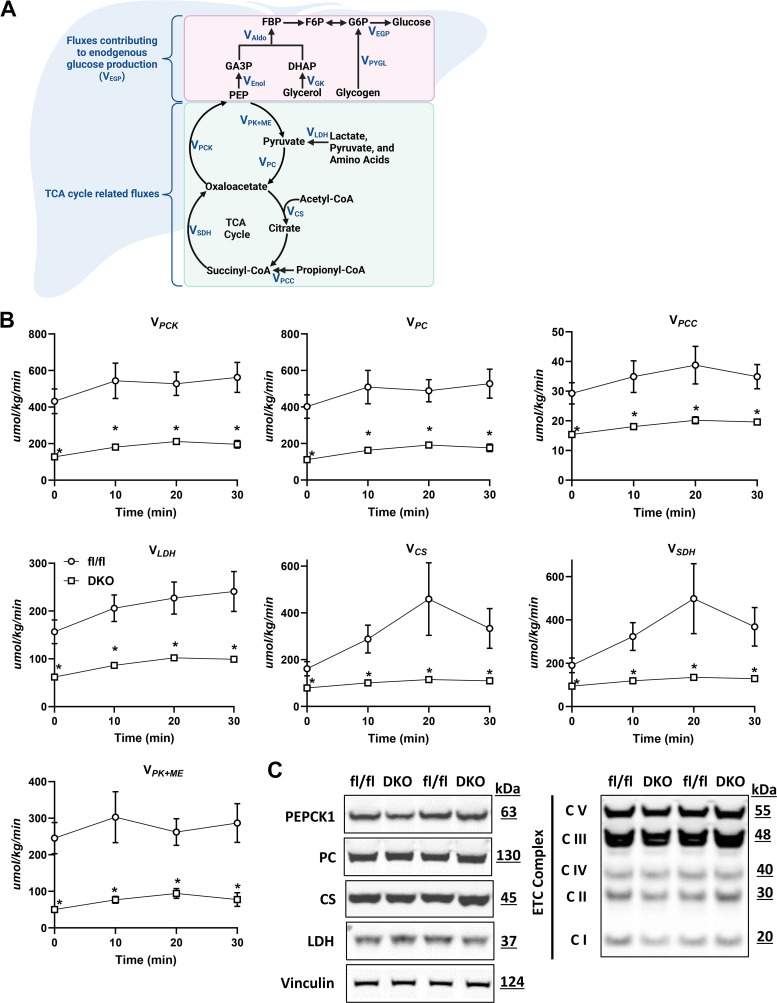
*A*: schematic representation of select glucose producing and tricarboxylic acid cycle fluxes quantified by ^2^H/^13^C metabolic flux analysis. Created using BioRender.com. *B*: model-estimated, nutrient fluxes (µmol/kg/min) in fl/fl and double knockout (DKO) mice prior to and during a 30-min of treadmill run for total cataplerosis (*V*_PCK_), anaplerosis from pyruvate (*V*_PC_), anaplerosis from propionyl-CoA (*V*_PCC_), flux from unlabeled, non-phosphoenolpyruvate, anaplerotic sources to pyruvate (*V*_LDH_), flux from oxaloacetate and acetyl-CoA to citrate (*V*_CS_), flux from succinyl-CoA to oxaloacetate (*V*_SDH_), and pyruvate cycling (*V*_PK + ME_). Data are means ± SE (*n* = 8 mice each group). *P < 0.05 vs. fl/fl at specified time point by two-way repeated-measures ANOVA followed by Šidák’s post hoc tests. *C*: representative Western blots using liver lysates from fl/fl and DKO mice after completing this exercise protocol using the indicated antibodies.

In addition to glucose fluxes, mitochondrial oxidative fluxes were determined in WT and DKO mice (Fig. 6*A*). Consistent with the lower gluconeogenesis from PEP, cataplerosis (*V*_PCK_) was decreased in DKO mice at rest and during exercise (Fig. 6*B*). Anaplerosis and related fluxes (*V*_PC_, *V*_PCC_, *V*_LDH_) were lower at rest and during exercise in DKO mice compared with WT mice (Fig. 6*B*). This included the flux of non-phosphoenolpyruvate anaplerotic sources to pyruvate (*V*_LDH_), which encompasses lactate and alanine conversion to pyruvate. TCA cycle fluxes (*V*_CS_ and *V*_SDH_) and pyruvate cycling (*V*_PK + ME_) increased during exercise in WT mice (Fig. 6*B*). However, the rise in these fluxes was markedly abrogated in DKO mice. Together, these results show that inhibiting key nodes regulating pyruvate entry and generation in the mitochondria impairs the oxidative and gluconeogenic capacity of the liver and limits the ability to the liver to meet the glucose demands of working muscle.

We assessed the protein expression of the enzymes catalyzing key reactions in these pathways, including phosphoenolpyruvate carboxykinase 1 (PEPCK1), pyruvate carboxylase (PC), citrate synthase (CS), and lactate dehydrogenase (LDH) in liver samples collected from these mice at the end of the study. Although flux through these metabolic pathways was markedly suppressed, the protein abundance of these enzymes was not affected in liver of the DKO mice compared with WT controls (Fig. 6*C*). In addition, the expression of subunits of the five electron transport chain complexes was also not affected by loss of ALT2 and the MPC, suggesting that reduced flux through these metabolic reactions is due to impaired pyruvate and alanine metabolism.

## DISCUSSION

Accelerated hepatic glucose production via glycogenolysis and gluconeogenesis during exercise plays an important role in fueling muscle contraction via the activities of the Cori and Cahill cycles. To evaluate the role of hepatic mitochondrial pyruvate and amino acid metabolism, we tested exercise performance and metabolic responses to acute exercise in mice lacking MPC2, ALT2, or both proteins in a hepatocyte-specific manner. We found that deletion of either MPC2 or ALT2 alone did not significantly impair exercise performance, but that DKO mice fatigued sooner and were hypoglycemic during and after the exercise challenge. Moreover, using sophisticated ^2^H/^1^³C metabolic flux analyses we demonstrated that DKO mice exhibited lower endogenous glucose production at rest and during exercise (owing to a decrease in both glycogenolysis and gluconeogenesis). The decline in gluconeogenesis was linked to decreased anaplerotic, cataplerotic, and TCA cycle fluxes. These data suggest that metabolic inhibition of both the Cori and Cahill cycles is required to jointly lower hepatic glucose production and constrain exercise performance in mice.

The liver has an extraordinary ability to extract multiple types of nutrients from the blood and channel them to gluconeogenesis, which allows the liver to effectively maintain glucose homeostasis and meet the glucose demands of working muscle. The data presented herein highlight the important roles that coordinated use of different fuel sources in liver mitochondrial metabolism plays in maintaining blood glucose concentrations during exercise and how this may impact prolonged exercise performance. The present findings demonstrate that only in the context of preventing the use of multiple gluconeogenic precursors (i.e., a major interruption in the ability of the liver to transition to the gluconeogenic mode) that accelerated gluconeogenesis during exercise and exercise performance are impacted.

Previously we have shown that the modest effects of deleting the MPC in the liver on blood glucose under fasted conditions were mitigated, at least in part, by compensatory pyruvate-alanine cycling via ALT2 (18). In other work, disruption of the gene encoding ALT2 (Gpt2) in hepatocytes resulted in little discernable effect on glucose homeostasis in fasted lean mice and this is likely due to alanine-pyruvate cycling via the intact MPC in those mice (24). Similarly, liver-specific deletion of either *Mpc2* or *Gpt2* had no effect on blood glucose concentrations after exercise. However, we show that combined loss of *Gpt2* and *Mpc2* leads to marked impairment in hepatic gluconeogenic, cataplerotic, anaplerotic, and overall TCA flux especially during treadmill exercise. The phenotype of these mice during exercise is quite consistent with recently reported effects of deleting hepatic PEPCK1 (5, 40), which also impairs flux of pyruvate and alanine into gluconeogenic pathways. Interestingly, we have found that DKO mice can maintain normal blood glucose during 16-h fasted conditions even with concomitant liver-specific suppression of glycerol-mediated gluconeogenesis (26). This is likely due to the gluconeogenic contributions of other tissues (kidney and small intestine) under chronic fasted conditions (16, 41, 42). The inability of DKO mice to maintain normoglycemia during exercise highlights the tremendous demand for peripheral glucose supply during exercise and the potential inability for other gluconeogenic tissues to compensate in the context of an acute and robust demand for hepatic gluconeogenesis.

Results from the metabolic flux studies showed that loss of both MPC and ALT2 resulted in reduced gluconeogenesis at rest and attenuated the increased gluconeogenesis with the onset of exercise. Interestingly, gluconeogenesis from glycerol began to increase in DKO mice after 20 min of continuous aerobic activity. There are two pathways for gluconeogenesis from glycerol. The direct pathway reverses some steps of glycolysis whereas the indirect pathway requires mitochondrial metabolism of glycerol as pyruvate. Our recent work has demonstrated that loss of the MPC impairs glycerol metabolism through the indirect pathway, but does not affect overall glycerol-mediated gluconeogenesis, which is the predominant pathway in liver (26). However, increased gluconeogenesis from glycerol was not sufficient to compensate for the dramatic reductions in gluconeogenesis from pyruvate/lactate and alanine in the DKO, as the absolute contribution of glycerol to overall glucose production was relatively low (Fig. 5*E*). This resulted in reduced blood glucose and elevated blood lactate concentrations during and after exercise. Reductions in blood glucose and accumulation of blood lactate beyond the rate of clearance correlate well with exercise fatigue during fasting (43), which could explain reduced performance in DKO mice, though we did not perform experiments to determine the relative roles of each in the present study.

A graded intensity treadmill running bout (plateaued at 30 m/min) revealed that DKO mice exhausted more quickly than control mice suggestive of reduced exercise endurance. Liver glycogen levels are positively associated with time to exhaustion during exercise in mice and humans (44, 45), suggesting that liver glycogenolysis is an important fuel source for exercise endurance. We could not determine liver glycogen content dynamically prior to and during exercise. However, since sedentary DKO mice exhibited diminished liver glycogen compared with WT littermates, it is likely that the DKO mice rely heavily on hepatic glycogenolysis and thus deplete this fuel source very quickly when food is not present. In addition, infusion of glucose prior to exercise may have affected and altered pre-exercise hepatic glycogen content differently in exercised DKO versus sedentary controls, which is another caveat to these conclusions.

Similar to the DKO mice in this study, inhibiting liver gluconeogenesis by deleting PEPCK1 lowers time to exhaustion in mice running at a submaximal running speed (40). Taken with the current study, these results support historical literature that liver glucose production from glycogenolysis and/or gluconeogenesis is an important contributor to exercise endurance. This is not surprising given that even a low-to-moderate intensity bout of treadmill running at 12 m/min (the same as that performed during our isotope infusion studies) increases skeletal muscle glucose clearance and depletes skeletal muscle glycogen in mice (46). In doing so, the liver becomes crucial in supplying glucose to meet the energy demands of working muscle. On the other hand, the maximal running speed obtained during an exercise stress test in the DKO mice was not different from that of WT control mice (Fig. 5*B*). The comparable maximal running speed between WT and DKO mice may be due to the maximal running speed exercise test being cardiopulmonary-limited as it is near maximal whole body oxygen utilization (47). It also could be due to differences in fuel (fatty acids or muscle and liver glycogen) utilization between moderate and maximal running speed. Thus, the differences in the availability of glucose and associated nutrients that occur in DKO mice are not likely to limit aerobic higher-intensity exercise capacity that is near maximum capacity.

It is important to note that mice used for exercise experiments only engaged in acute bouts of exercise, and how inhibition of MPC, ALT2, or both affects adaptations to chronic exercise training remains unexplored. However, recent work with another model with liver-specific defects in gluconeogenesis, the liver-specific PEPCK knockout mouse, revealed no defects in adaptations to exercise training (5).

In conclusion, combined deletion of Mpc2 and Gpt2 in liver of mice markedly impairs hepatic gluconeogenesis and leads to hypoglycemia, hyperlactatemia, and reduced exercise performance in a graded intensity treadmill protocol. Moreover, loss of mitochondrial pyruvate and alanine metabolism results in markedly reduced flux through a variety of mitochondrial pathways in liver during exercise. These findings highlight the importance of hepatic glucose production in many exercise paradigms.

## DATA AVAILABILITY

Data will be made available upon reasonable request.

## SUPPLEMENTAL DATA

10.6084/m9.figshare.25035806.v1 Supplemental Material (Supplemental Tables S1–S3) including additional methodological details and uncut Western blots: https://doi.org/10.6084/m9.figshare.25035806.v1.

## GRANTS

This work was funded by NIH Grants R01 DK104735 (to B.N.F) and R01 DK117657 (to B.N.F.). The Core services of the Diabetes Research Center under Grant No. P30 DK020579 and the Nutrition Obesity Research Center under Grant No. P30 DK56341 at the Washington University School of Medicine also supported this work. D.F. was supported by an NIH training grant and later by a K01 Awards T32 DK007120 and K01 DK137050. R.T.B. was supported by K01 HL145326. J.P.T. was supported by NIH Grants R01 DK121497, R01 AG069781, U01 AG070928, and VA 1I01BX002567. Metabolic flux analyses were performed by the Vanderbilt Mouse Metabolic Phenotyping Center under Grant Nos. U2C DK135073 and P60 DK020593.

## DISCLOSURES

B.N.F. is a shareholder and a member of the Scientific Advisory Board for Cirius Therapeutics, which is developing an MPC modulator for treating nonalcoholic steatohepatitis. None of the other authors has any conflicts of interest, financial or otherwise, to disclose.

## AUTHOR CONTRIBUTIONS

M.R.M., L.L., C.C.H., and B.N.F. conceived and designed research; M.R.M., M.H., D.F., L.L., and C.C.H. performed experiments; M.R.M., M.H., D.F., L.L., and C.C.H. analyzed data; M.R.M., M.H., D.F., R.T.B., J.P.T., G.A.M., L.L., C.C.H., and B.N.F. interpreted results of experiments; M.R.M., M.H., D.F., L.L., C.C.H., and B.N.F. prepared figures; M.R.M., M.H., D.F., L.L., C.C.H., and B.N.F. drafted manuscript; M.R.M., M.H., D.F., R.T.B., J.P.T., G.A.M., L.L., C.C.H., and B.N.F. edited and revised manuscript; M.R.M., M.H., D.F., R.T.B., J.P.T., G.A.M., L.L., C.C.H., and B.N.F. approved final version of manuscript.
